# The Safety and Efficacy of Phage Therapy for Infections in Cardiac and Peripheral Vascular Surgery: A Systematic Review

**DOI:** 10.3390/antibiotics12121684

**Published:** 2023-11-30

**Authors:** Emily A. Simpson, Caitlin S. MacLeod, Helen J. Stacey, John Nagy, Joshua D. Jones

**Affiliations:** 1 Medical Microbiology, Ninewells Hospital, NHS Tayside, Dundee DD2 1SG, UK; 2 Department of Vascular Surgery, Ninewells Hospital, NHS Tayside, Dundee DD2 1SG, UK; 3 Division of Systems Medicine, School of Medicine, University of Dundee, Ninewells Hospital, Dundee DD2 1SG, UK; 4 Public Health, Kings Cross Hospital, Clepington Road, NHS Tayside, Dundee DD3 8EA, UK; 5 Infection Medicine, Edinburgh Medical School: Biomedical Sciences, University of Edinburgh, Chancellor’s Building, 49 Little France Crescent, Edinburgh EH16 4SB, UK

**Keywords:** bacteriophage, cardiac surgery, peripheral vascular surgery, phage therapy, systematic review

## Abstract

New approaches to managing infections in cardiac and peripheral vascular surgery are required to reduce costs to patients and healthcare providers. Bacteriophage (phage) therapy is a promising antimicrobial approach that has been recommended for consideration in antibiotic refractory cases. We systematically reviewed the clinical evidence for phage therapy in vascular surgery to support the unlicensed use of phage therapy and inform future research. Three electronic databases were searched for articles that reported primary data about human phage therapy for infections in cardiac or peripheral vascular surgery. Fourteen reports were eligible for inclusion, representing 40 patients, among which an estimated 70.3% of patients (n = 26/37) achieved clinical resolution. A further 10.8% (n = 4/37) of patients showed improvement and 18.9% (n = 7/37) showed no improvement. Six of the twelve reports that commented on the safety of phage therapy did not report adverse effects. No adverse effects documented in the remaining six reports were directly linked to phages but reflected the presence of manufacturing contaminants or release of bacterial debris following bacterial lysis. The reports identified by this review suggest that appropriately purified phages represent a safe and efficacious treatment option for infections in cardiac and peripheral vascular surgery.

## 1. Background

Complicating infections in cardiac and peripheral vascular surgery (cardiac/vascular surgery) occur in up to around 6% of cases, depending upon the site of infection, and are associated with mortality rates up to 75% [1,2]. The management of such infections includes aggressive surgery, with complete device/graft removal and reconstruction when possible. In many cases surgical management is not appropriate due to the high risk of morbidity or mortality and lengthy courses of antibiotics may be required [1,3]. As well as potentially devastating consequences for patients, such surgical site infections come at significant cost to healthcare providers. For example, vascular surgical site infections have been associated with between 4.8 and 24.0 excess inpatient bed days and notable lengths of readmission [4].

The causative microorganisms can be identified in 75–98% of cases. Gram-positive organisms, notably Enterococci and Staphylococci, have been reported to account for up to 58% of vascular graft infections, with Gram-negative organisms accounting for 34% [1]. However, the effectiveness of antibiotics is often considered to be hampered by biofilm formation, particularly on the surfaces of devices/grafts that cannot be removed [5]. Biofilms are extracellular polysaccharide matrices which can afford bacteria tolerance to antibiotics [6,7]. High rates of mortality may be encountered in patients managed with antibiotics [1,8]. Given the difficulties often encountered when managing vascular infections and the significant costs to both patients and healthcare systems, new approaches to managing these infections are urgently needed.

Bacteriophages (phages) are naturally occurring viruses that generally infect bacteria in a species-specific manner. Phage therapy was used widely in the 20th century before a lack of understanding of phages and the mass production of antibiotics curtailed its use, although not in the geopolitical East [9]. The antimicrobial resistance crisis has driven renewed Western interest in phage therapy, with an increasing number of phage therapy applications among patients with antibiotic refractory infections [10]. The American Antibiotic Resistance Leadership Group and Health Improvement Scotland have recently recommended that phage therapy be considered for antibiotic refractory infections [11,12]. There are compelling observational and trial data in favour of the safety of phage therapy [10,13], likely reflecting our co-evolution with phages. While observational data from patients with antibiotic refractory infections are compelling, it has proven challenging to demonstrate its efficacy through clinical trials. This likely reflects complexities in successfully employing phage therapy, rather than a mechanistic shortcoming. The two trials that have overcome these complexities and achieved the ‘Goldilocks’ constellation of factors required for a successful outcome have successfully shown evidence of efficacy [14,15]. Successful phage therapy requires the tailoring of both phages and clinical approach and requires the delivery of the right phage(s) at the right time to the site(s) of infection containing phage-susceptible bacterial cells [13].

Phage therapy is not currently licensed in any Western context. The unlicensed use of phage therapy typically requires a presentation of evidence supporting its use as part of health authority or local hospital governance. Many clinicians submitting to such governance mechanisms are unlikely to have experience in or in-depth knowledge of phage therapy. It is therefore important to collate data about phage therapy in specific specialties to support and facilitate applications for the unlicensed use of phage therapy and inform future research. Therefore, this systematic review will, without limitations on study design, collate reports of phage therapy for the treatment of cardiac/vascular surgery infections in humans and review the evidence base for the safety and efficacy of phage therapy in cardiac/vascular surgery.

## 2. Methods

### 2.1. Search Strategy

Three electronic databases were searched, without limits, for articles published up to March 2022: EMBASE (1980–2022), Ovid MEDLINE^®^ Epub Ahead of Print; In-Process & Other Non-Indexed Citations, Ovid MEDLINE^®^ Daily, Ovid MEDLINE and Versions^®^ (1946–2022); and Web of Science. The Web of Science Core Collection Citation Indexes searched were: Science Citation Index Expanded (1900–2022), Book Citation Index–Science (2005–2022), and the Emerging Sources Citation Index (2015–2022). The search was performed using the following terms: (bacteriophage* OR phage*) AND (sepsis OR heart OR prosthe* OR vascu* OR aort* OR septi* OR endocardi* OR ventricul* OR cardi* OR bacter?em* OR allograft* OR endograft* OR graft* OR valv* OR bypass OR aneurysm OR stent* OR EVAR) AND (clinic* OR trial OR treat* OR case* OR patient* OR therap*). The asterisk (*) represents any number of characters, including no character. In Ovid, these terms were followed by the suffix ‘.mp.’ and they were searched as topics on the Web of Science platform. This systematic strategy was supplemented using reports known to the authors that described the use of phage therapy for a wide variety of conditions but the titles and abstracts of which would not be detected by the specific search terms used [16], reports of interest identified from reference lists [17], and relevant papers that became available after the systematic search date [18,19,20,21,22,23,24]. A protocol was not published prior to this study.

### 2.2. Study Selection Criteria

The titles and abstracts of all the articles were screened for eligibility. Articles were included if they contained primary clinical data about the use of phage therapy in humans to treat any type of infection which could be seen in cardiac or peripheral vascular surgery. This included infections associated with the endocardium, ventricular assist devices, prosthetic valves, vascular grafts, surgical sites from vascular procedures, and sepsis from a vascular source. Articles had to be available in the English language. Secondary literature was excluded unless it reported primary clinical data unavailable from the primary source. There were no restrictions on study date, type, or location. There was no limitation on the purity of phage preparation used or route(s) of administration. Articles reporting the use of phage-derived products (e.g., endolysins) were excluded. Study selection was carried out independently by two authors (CSM, JDJ), with discrepancies resolved by discussion with an additional author (HJS). Deduplication was performed using Endnote (version X9.2). Title and abstract screening and subsequent full-text eligibility screening were performed independently by three authors (CSM, EAS, JDJ), with discrepancies resolved by discussion with an additional author (HJS). This review was conducted in accordance with the PRISMA (Preferred Reporting Items for Systematic Reviews and Meta-Analyses) guidelines [25], and a PRISMA checklist was completed [26] (Supplementary files S1 and S2).

### 2.3. Data Extraction and Critical Appraisal

The following information was extracted from each eligible study: author(s); date of publication; study location; study type; number of relevant reports; condition microbiology; clinical condition and when possible, patient age(s) and/or previous treatment(s); details of the phage treatment; treatment schedule and route(s), including details of other ongoing therapies (e.g., antibiotics) when reported; treatment efficacy; and comments or data regarding safety and adverse effects. When possible, the cases reported by included reports were classified as ‘resolved’, ‘improved’, or ‘no response’. The term ‘resolved’ was defined as clinical resolution of infection. The extraction of data from each eligible article was performed independently by two authors (CSM, JDJ, or EAS), with discrepancies resolved by discussion with an additional author (HJS). All eligible studies were assessed using the appropriate Joanna Briggs Institute critical appraisal checklist [27]; Supplementary file S3. The critical appraisal was performed independently by two authors (JDJ, CSM, or EAS), with discrepancies resolved by discussion with an additional author (HJS). The influence of publication bias and selective reporting on the cumulative evidence are considered in the discussion. Fisher’s exact test was performed using an online GraphPad^®^ tool [28].

## 3. Results

**Figure 1:**
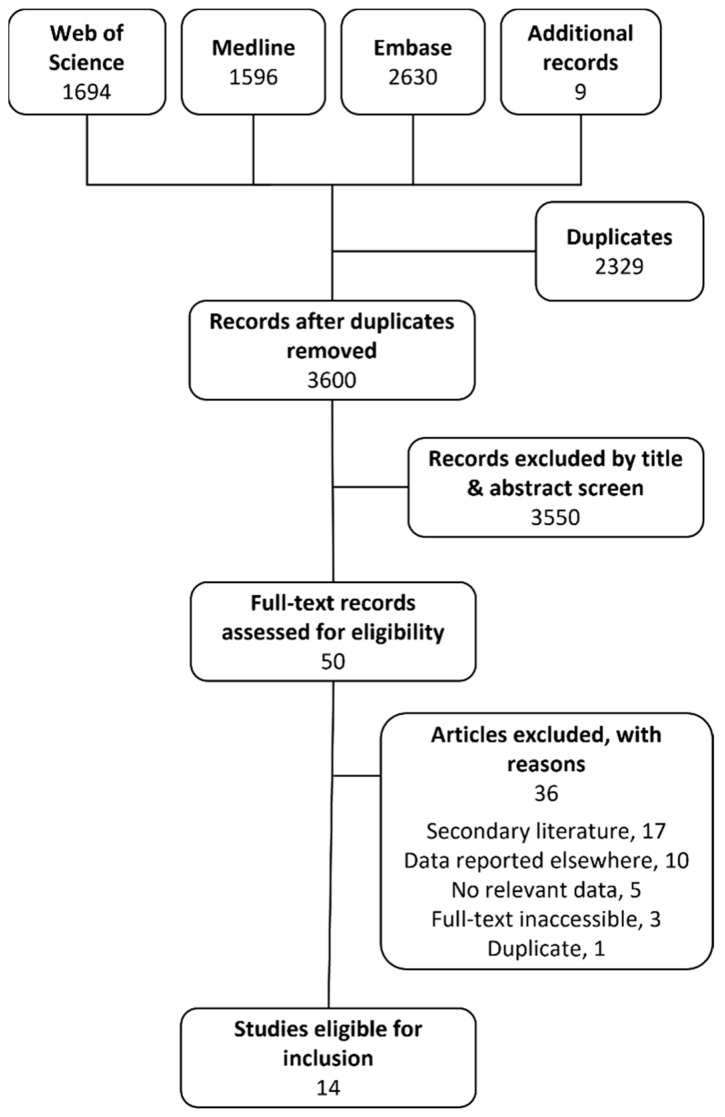
Flow diagram of study selection.

The systematic search identified 5920 records. After duplicates were removed, 3591 records remained, published between 1931 and 2022. Nine additional records were identified from other sources. One report described the use of phage therapy for a wide variety of conditions and therefore the title and abstract were not detected by the specific search terms used [16], an undetected report of potential interest was identified from secondary literature references [17], and we became aware of seven further relevant records published after the systematic search date which were included for completeness [18,19,20,21,22,23,24]. Title and abstract screening led to the exclusion of 3550 records that did not meet the inclusion criteria (Figure 1). Of the remaining 50 articles eligible for full-text screening, 36 were excluded because they were secondary literature (n = 17), contained data reported elsewhere (n = 10; this included papers which nonetheless provided additional information regarding included cases [29,30,31,32]), did not contain relevant data (n = 5), were not available in full (n = 3), or were a duplicate record (n = 1).

A total of 14 eligible studies were identified for inclusion in this review [16,18,19,20,21,22,23,24,33,34,35,36,37,38]. The data extracted from these studies are shown in Supplementary file S4. These studies were from Germany (n = 5), the United States (n = 4), Australia (n = 1), Israel (n = 1), Latvia (n = 1), Poland (n = 1), and Spain (n =1). There were seven case series and seven case reports. Critical appraisal highlighted various shortcomings in the quality of reporting but did not provide evidence of bias warranting the exclusion of any of the studies (Supplementary file S2).

Together the 14 records reported data regarding 40 patients treated for: ventricular-assist-device-associated infections (n = 15); ‘pyopericardium’ (n = 7); prosthetic valve-related infective endocarditis (n = 5); infective endocarditis (n = 3); aortic graft infection: aortic arch replacement (n = 3), anatomical region of aortic graft unspecified (n = 1), and TEVAR (n = 1); ascending aorta-associated infection (unclear if the graft was in situ; n = 1); peripheral vascular graft infection (axillo-bifemoral bypass; n = 1); CIED (cardiac implantable electronic device) and carotid subclavian bypass infection (n = 1); trepostinil pump infection (n = 1); and sternal wall healing (n = 1). The bacteria most commonly targeted by phage therapy were of the genera *Pseudomonas* (described in 10 of 14 reports) or *Staphylococcus aureus* or other Staphylococcal species (described in nine of 14 included reports). Other bacteria targeted included *E. coli*, *Enterococcus faecium, Proteus mirabilis,* and *Cutibacterium acnes*. Infections were described as refractory to antibiotics in 12 of the 14 articles; antibiotic sensitivities were not reported by Fabijan and colleagues and it was unclear from Püschel and colleagues whether the bacteria they were targeting were refractory to antibiotic therapy [21,36].

Nine of the 14 articles reported the use of a phage cocktail to treat patients, two articles reported the use of only one type of phage, two articles reported the use of both monophage preparations and cocktails in various patients, and the details of the phage(s) used were not clear in one article. Prospective screening of bacterial susceptibility to phage therapy was reported by eight of 14 articles, three articles did not report testing, and it was unclear whether testing was undertaken pro- or retrospectively in two articles. Sensitivity testing by Slopek and colleagues was implied by an earlier, related, publication [39]. Phage therapy was most often administered directly into the site of infection during or after surgery (n = 9/14), supplemented by intravenous phage therapy for some patients in three reports. Pre-administration rinsing with a weak bicarbonate solution, described in some phage cases in the literature, was not reported [40]. Intravenous phage therapy alone was described in three reports. Oral use was described in two reports, preceded by gastric neutralisation in one report. The use of antibiotics in combination with phage therapy was reported by 13 of the 14 articles; it was unclear if concurrent antibiotic therapy was used by Slopek and colleagues.

A precise efficacy estimate cannot be derived, as co-administered therapies, phages, reporting timepoints, and methodologies differed among the articles. Notwithstanding these caveats and given that most infections reported were refractory to antibiotics, a crude and cautionary estimate of efficacy can be derived. Together, the 14 articles represented the treatment of 40 cardiac/vascular surgery patients. There was insufficient information to confidently classify the outcome for three patients. Among the 37 patients for whom there was sufficient evidence to draw a conclusion about the efficacy of phage therapy, 70.3% (n = 26/37) achieved resolution of their infection, 10.8% (n = 4/37) showed improvement, and 18.9% (n = 7/37) showed no improvement.

We investigated whether the prospective screening of bacterial susceptibility to the phages used affected clinical outcomes. The eight articles that reported prospectively screening bacterial susceptibility to the phages represented 21 patients, among which 57.1% (n = 12/21) achieved resolution of their infection, 14.3% (n = 3/21) showed improvement, and 28.6% (n = 6/21) had no response. In contrast, the six articles which did not explicitly report prospectively screening bacterial susceptibility represented 16 patients, among which 87.5% (n = 14/16) achieved resolution of their infection, 6.3% (n = 1/16) showed improvement, and 6.3% (n = 1/16) had no response. Prospectively screening bacterial susceptibility to the phages made no significant difference to the proportion of patients that achieved resolution (*p* = 0.071) or resolution/improvement (*p* = 0.113).

Among the 12 of 14 articles which commented on safety or adverse effects, no adverse effects were reported by six articles [19,20,22,24,34,36]. Of the remaining six articles, two did not comment specifically about the particular patients included in this review. Slopek and colleagues generally remarked that side effects were rarely encountered but did not comment on specific patients [16]. Similarly, Onallah and colleagues reported that ‘no major side effects were reported’ in their cohort and no explanations were suggested for the headaches (one patient) or tingling at the infection site (two patients) reported during treatment [23].

Four articles reported possible adverse effects. While Rubalskii and colleagues reported ‘no adverse effects’, they noted that six of eight patients had elevated CRP levels shortly after phage therapy, which could be potentially attributed to normal postoperative conditions or a consequence of bacterial lysis [37]. Duplessis and colleagues could not rule out endotoxin release as a contributing factor in their patient’s decompensation 36 h after phage administration, although this was considered to primarily reflect the ‘frank progression of untreated fluid collections, antecedent influenza infection and end-stage cardiac failure’ [35]. While no adverse events were reported for three of the four patients included from Aslam and colleagues’ case series, one patient developed a fever, wheezing, and shortness of breath about two hours after each of the two first consecutive intravenous infusions of the phage cocktail. However, the cocktail was subsequently used for the remainder of the course at a 10-fold lower dose without adverse effects. Aslam and colleagues reported that the endotoxin level of the phage preparation was acceptable and hypothesised that unknown additional pyrogens may have been present in the phage preparation that were sufficiently diluted at the lower concentration [33]. In Tkhilaishvili and colleagues’ case series of four patients, nausea was reported for two patients and mild liver function test elevation in one patient [38]. The case of one patient who received intraoperative phage therapy and subsequently reported nausea had been previously published [30]; it was unclear which of the other three patients experienced nausea. No explanation was suggested for the nausea. The case of the patient who exhibited mild liver function test elevation had been previously published elsewhere [31]. The phage preparations used were reported to be within the recommended limit for endotoxin administration. Notably, a 0.5 log reduction in bacteriophage dose permitted the continuation of intravenous phage therapy and normalisation of laboratory values.

## 4. Discussion

The evidence collated by this systematic review suggests that phage therapy is a highly effective treatment option for infections in cardiac/vascular surgery, with 70.3% of patients achieving resolution of infection. Of course, while the evidence in favour of phage therapy is compelling in these antibiotic refractory cases, especially in the context of in vitro phage susceptibility data, the relative contributions of simultaneous further surgical interventions, antibiotics, and phage therapy cannot be delineated. However, as noted by Racenis and colleagues, the combination of surgery, antibiotics, and phages has great potential for resolving infections in cardiac/vascular surgery [20].

Successful phage therapy relies on delivering enough phages to which the bacteria are sensitive to the site(s) of infection at the right time during the course of the infection. Treatment failure or partial success may occur if this is not achieved. In the case of a 51-year-old male with a chronic LVAD infection reported by Rubalskii and colleagues, a 100× reduction in *S. aureus* in drainage fluid was identified but the patient declined further surgical intervention to improve the delivery of phages to the infected sites and the patient died from *S. aureus* sepsis 1.5 months after starting phage therapy [37]. Although phage therapy is currently only available on an unlicensed basis, the timing of administration within the clinical course of infection requires consideration, with late administration risking the infection being irretrievable despite phage therapy. However, in some cases, such as that reported by Duplessis and colleagues, co-morbidities and co-infections can accelerate or be primarily responsible for deterioration. In this complex case, although phage therapy appeared to achieve sterile blood cultures, the patient’s deterioration and death were attributed to the ‘frank progression of untreated fluid collections, antecedent influenza infection and end-stage cardiac failure’. The time lag between concluding that phage therapy was appropriate and its subsequent administration was unclear; it is therefore not possible to conclude whether an earlier administration of phage therapy may have been beneficial [35]. Treatment success can also be prevented by the development of bacterial resistance to the phages used or neutralising immune responses against phages, although the latter does not always preclude a successful outcome [41,42]. Bacterial resistance to phages was observed in two of the seven patients identified by this review who did not respond to phage therapy [24,38]. The development of bacterial resistance to phages can be associated with fitness costs for the bacteria; for example, a greater susceptibility to antibiotics post phage therapy was reported by Blasco and colleagues [24,43]. Secondary infections, caused by either unknown pre-existing or newly acquired bacteria, can also complicate outcomes. For example, Rubalskii and colleagues reported that in one patient, the causative bacteria (*S. aureus*, *P. aeruginosa,* and *E. faecium*) were not detected for 16 days after the last application of phage therapy, but the patient subsequently died due to an infection caused by *E. coli* and *P. aeruginosa*. It was unclear whether the secondary *P. aeruginosa* was the same as that treated with phage therapy, but the authors suggested this was not the case, as the antibiotic sensitivity profiles of the two strains were different [37]. However, in some cases, the reasons for treatment failure may remain unclear [16,22,23,33,36]. The prospective screening of bacterial susceptibility to the phages used is broadly considered essential [44]. That this review found that the prospective screening of bacterial susceptibility made no significant difference to outcomes likely reflects incomplete reporting. Although the proportion of patients achieving resolution of infection was greater among the studies that did not report prospective screening, that clinical resolution was achieved suggests that prospective screening may have been performed but not explicitly reported.

The evidence about the safety of phage therapy from the reports included in this review echoes the compelling broader clinical trial and observational evidence summarised elsewhere [10,13]. When adverse effects were reported, these were not linked to the phages themselves, but were considered to reflect the action of phages on bacteria or the presence of manufacturing contaminants. Adverse effects linked to bacterial lysis or the manufacturing contaminants have been reported elsewhere [40,45,46].

The reports included in this review show wide variation in clinical protocols. For example, Chan and colleagues achieved resolution of an aortic graft infection with a single local administration via a fistula under computed tomography guidance, while Rojas and colleagues achieved resolution with a single intraoperative application of phages mixed with a viscous galenic, and Rubalskii and colleagues achieved resolution with a single application of phages mixed with fibrin glue [18,34,37]. In contrast, other reports employed intravenous phage therapy of various doses and durations, often in combination with local administration [20,22,33]. Inter-practitioner variation likely reflects different independent approaches to solving similar clinical challenges and novel approaches can be important in driving ingenuity. In contrast, intra-practitioner variation reflects attempts to address patient-specific factors. Such variation is to be expected as approaches to achieving the ‘Goldilocks’ constellation of factors required for a successful outcome will require tailoring clinical approaches to individual patients.

Although comprehensive, the scope of this review is limited by its reliance on online English language sources. Phage therapy has a lengthy history of use, particularly in Russia and Georgia, and evidence relevant to this review may not have been available, accessible, or indexed in the databases searched. For example, a potentially relevant report from France in 1978 discussing the treatment of endocarditis caused by *Serratia* was inaccessible [47]. This review may also be limited by publication bias, with successful rather than unsuccessful phage therapy cases perhaps more likely to be published. Observational data may also be subject to selective reporting, suppressing negative findings. Critical appraisal of papers is important to assess the impacts of such biases on confidence in the body of evidence and to highlight incomplete reporting. Although the risks of such biases are inherent in observational data, confidence can be drawn from the consistently similar findings reported by different groups. While every effort was made to design a comprehensive search strategy, systematic reviews are inherently limited to the identification of reports matching the search criteria, and it is possible that relevant evidence from reports not matching the search criteria may have been omitted. Moreover, systematic reviews demand a defined search date, and, while additional relevant evidence published during study completion may be included, it is challenging for a methodical systematic review process to remain up to date in such a fast-moving field.

## 5. Conclusions

In summary, this review suggests that phage therapy is a safe and effective intervention for antibiotic refractory infections in cardiac/vascular surgery. The safety and efficacy findings from this review are in line with those of broader systematic reviews of phage therapy [10]. There is significant ongoing interest in the use of phage therapy for infections in cardiac/vascular surgery, with information about 13 of the 40 patients included (33%) published in the last two years. Resolution of antibiotic refractory infections in cardiac/vascular surgery with unlicensed phage therapy promises to reduce morbidity and mortality while delivering cost-savings for health authorities. Future use of licensed phage products could help prevent the development of such infections.

## Data Availability

Data sharing is not applicable to this article as no datasets were generated or analysed during the current study.

## Supplementary Materials

The following supporting information can be downloaded at: https://www.mdpi.com/article/10.3390/antibiotics12121684/s1, Supplementary file S1. PRISMA abstract checklist. Supplementary file S2. PRISMA checklist. Supplementary file S3. Critical appraisal. Supplementary file S4. Data extracted from included reports.

## Abbreviations

BD	twice daily;
CIED	cardiac implantable electronic device;
CRP	C-reactive protein;
CT	computed tomography;
EVAR	endovascular aortic repair;
LVAD	left ventricular assist device;
MSSA	methicillin-susceptible *Staphylococcus aureus*;
PET-CT	positron emission tomography-computed tomography;
PFU	plaque forming unit;
PRISMA	Preferred Reporting Items for Systematic Reviews and Meta-Analyses;
QID	four times daily;
TDS	three times daily;
TEVAR	thoracic endovascular aortic repair;
WBC	white blood cell count.
