# Supplementary material for: The Safety and Efficacy of Phage Therapy for Infections in Cardiac and Peripheral Vascular Surgery: A Systematic Review
Source: Antibiotics (Basel). 2023 Nov 30;12(12):1684. doi: 10.3390/antibiotics12121684 (PMC10740750; doi:10.3390/antibiotics12121684)
Supplement: Supplementary file 1 [file antibiotics-12-01684-s001.zip › antibiotics-2669945-supplementary.pdf]

Supplementary file S1: PRISMA 2020 for Abstracts Checklist

Section and Topic	Item #	Checklist item	Reported (Yes/No)
**TITLE**			
Title	1	Identify the report as a systematic review.	Y
**BACKGROUND**			
Objectives	2	Provide an explicit statement of the main objective(s) or question(s) the review addresses.	Y
**METHODS**			
Eligibility criteria	3	Specify the inclusion and exclusion criteria for the review.	N Insufficient space.
Information sources	4	Specify the information sources (e.g. databases, registers) used to identify studies and the date when each was last searched.	N Insufficient space.
Risk of bias	5	Specify the methods used to assess risk of bias in the included studies.	N Insufficient space.
Synthesis of results	6	Specify the methods used to present and synthesise results.	N/A
**RESULTS**			
Included studies	7	Give the total number of included studies and participants and summarise relevant characteristics of studies.	Y
Synthesis of results	8	Present results for main outcomes, preferably indicating the number of included studies and participants for each. If meta-analysis was done, report the summary estimate and confidence/credible interval. If comparing groups, indicate the direction of the effect (i.e. which group is favoured).	Y
**DISCUSSION**			
Limitations of evidence	9	Provide a brief summary of the limitations of the evidence included in the review (e.g. study risk of bias, inconsistency and imprecision).	N Insufficient space.
Interpretation	10	Provide a general interpretation of the results and important implications.	Y
**OTHER**			
Funding	11	Specify the primary source of funding for the review.	N/A

Section and Topic	Item #	Checklist item	Reported (Yes/No)
Registration	12	Provide the register name and registration number.	N/A

From: Page MJ, McKenzie JE, Bossuyt PM, Boutron I, Hoffmann TC, Mulrow CD, et al. The PRISMA 2020 statement: an updated guideline for reporting systematic reviews. BMJ 2021;372:n71. doi: 10.1136/bmj.n71
For more information, visit: http://www.prisma-statement.org/

Supplementary file S2: PRISMA 2020 Checklist

Section and Topic	Item #	Checklist item	Location where item is reported
**TITLE**			
Title	1	Identify the report as a systematic review.	Title
**ABSTRACT**			
Abstract	2	See the PRISMA 2020 for Abstracts checklist.	Abstract
**INTRODUCTION**			
Rationale	3	Describe the rationale for the review in the context of existing knowledge.	Background, paragraphs 1-4
Objectives	4	Provide an explicit statement of the objective(s) or question(s) the review addresses.	Background, paragraph 4
**METHODS**			
Eligibility criteria	5	Specify the inclusion and exclusion criteria for the review and how studies were grouped for the syntheses.	Methods, paragraph 2
Information sources	6	Specify all databases, registers, websites, organisations, reference lists and other sources searched or consulted to identify studies. Specify the date when each source was last searched or consulted.	Methods, paragraph 1
Search strategy	7	Present the full search strategies for all databases, registers and websites, including any filters and limits used.	Methods, paragraph 1
Selection process	8	Specify the methods used to decide whether a study met the inclusion criteria of the review, including how many reviewers screened each record and each report retrieved, whether they worked independently, and if applicable, details of automation tools used in the process.	Methods, paragraph 2
Data collection process	9	Specify the methods used to collect data from reports, including how many reviewers collected data from each report, whether they worked independently, any processes for obtaining or confirming data from study investigators, and if applicable, details of automation tools used in the process.	Methods, paragraph 3
Data items	10a	List and define all outcomes for which data were sought. Specify whether all results that were compatible with each outcome domain in each study were sought (e.g. for all measures, time points, analyses), and if not, the methods used to decide which results to collect.	Methods, paragraph 3
	10b	List and define all other variables for which data were sought (e.g. participant and intervention characteristics, funding sources). Describe any assumptions made about any missing or unclear information.	
Study risk of bias assessment	11	Specify the methods used to assess risk of bias in the included studies, including details of the tool(s) used, how many reviewers assessed each study and whether they worked independently, and if applicable, details of automation tools used in the process.	Methods, paragraph 3
Effect measures	12	Specify for each outcome the effect measure(s) (e.g. risk ratio, mean difference) used in the synthesis or presentation of results.	N/A

Section and Topic	Item #	Checklist item	Location where item is reported
Synthesis methods	13a	Describe the processes used to decide which studies were eligible for each synthesis (e.g. tabulating the study intervention characteristics and comparing against the planned groups for each synthesis (item #5)).	N/A
	13b	Describe any methods required to prepare the data for presentation or synthesis, such as handling of missing summary statistics, or data conversions.	N/A
	13c	Describe any methods used to tabulate or visually display results of individual studies and syntheses.	N/A
	13d	Describe any methods used to synthesize results and provide a rationale for the choice(s). If meta-analysis was performed, describe the model(s), method(s) to identify the presence and extent of statistical heterogeneity, and software package(s) used.	N/A
	13e	Describe any methods used to explore possible causes of heterogeneity among study results (e.g. subgroup analysis, meta-regression).	N/A
	13f	Describe any sensitivity analyses conducted to assess robustness of the synthesized results.	N/A
Reporting bias assessment	14	Describe any methods used to assess risk of bias due to missing results in a synthesis (arising from reporting biases).	Discussion, paragraphs 2-3
Certainty assessment	15	Describe any methods used to assess certainty (or confidence) in the body of evidence for an outcome.	Discussion, paragraphs 2-3
**RESULTS**			
Study selection	16a	Describe the results of the search and selection process, from the number of records identified in the search to the number of studies included in the review, ideally using a flow diagram.	Results, paragraph 1; figure 1
	16b	Cite studies that might appear to meet the inclusion criteria, but which were excluded, and explain why they were excluded.	Results, paragraph 1
Study characteristics	17	Cite each included study and present its characteristics.	Results, paragraphs 2-8; supplementary file 4
Risk of bias in studies	18	Present assessments of risk of bias for each included study.	Supplementary file 3
Results of individual studies	19	For all outcomes, present, for each study: (a) summary statistics for each group (where appropriate) and (b) an effect estimate and its precision (e.g. confidence/credible interval), ideally using structured tables or plots.	Supplementary file 4
Results of syntheses	20a	For each synthesis, briefly summarise the characteristics and risk of bias among contributing studies.	N/A
	20b	Present results of all statistical syntheses conducted. If meta-analysis was done, present for each the summary estimate and its precision	N/A

Section and Topic	Item #	Checklist item	Location where item is reported
		(e.g. confidence/credible interval) and measures of statistical heterogeneity. If comparing groups, describe the direction of the effect.	
	20c	Present results of all investigations of possible causes of heterogeneity among study results.	N/A
	20d	Present results of all sensitivity analyses conducted to assess the robustness of the synthesized results.	N/A
Reporting biases	21	Present assessments of risk of bias due to missing results (arising from reporting biases) for each synthesis assessed.	Discussion, paragraph 5
Certainty of evidence	22	Present assessments of certainty (or confidence) in the body of evidence for each outcome assessed.	Discussion, paragraph 5; supplementary file 3
**DISCUSSION**			
Discussion	23a	Provide a general interpretation of the results in the context of other evidence.	Discussion paragraphs 1-4
	23b	Discuss any limitations of the evidence included in the review.	Discussion, paragraphs 1, 5
	23c	Discuss any limitations of the review processes used.	Discussion, paragraphs 5
	23d	Discuss implications of the results for practice, policy, and future research.	Conclusions
**OTHER INFORMATION**			
Registration and protocol	24a	Provide registration information for the review, including register name and registration number, or state that the review was not registered.	Methods paragraph 1
	24b	Indicate where the review protocol can be accessed, or state that a protocol was not prepared.	Methods paragraph 1
	24c	Describe and explain any amendments to information provided at registration or in the protocol.	N/A
Support	25	Describe sources of financial or non-financial support for the review, and the role of the funders or sponsors in the review.	Funding statement
Competing interests	26	Declare any competing interests of review authors.	Conflict of interest statement
Availability of data, code and	27	Report which of the following are publicly available and where they can be found: template data collection forms; data extracted from included studies; data used for all analyses; analytic code; any other materials used in the review.	Data availability

Section and Topic	Item #	Checklist item	Location where item is reported
other materials			statement

From: Page MJ, McKenzie JE, Bossuyt PM, Boutron I, Hoffmann TC, Mulrow CD, et al. The PRISMA 2020 statement: an updated guideline for reporting systematic reviews. BMJ 2021;372:n71. doi: 10.1136/bmj.n71

For more information, visit: http://www.prisma-statement.org/

Supplementary file S3: critical appraisal

The critical appraisal of the manuscripts was performed using the Joanna Briggs Institute suite of critical appraisal tools available from https://jbi.global/critical-appraisal-tools (accessed on 06/07/2023). The case series tool was adapted to include comments on adverse effects and, when appropriate, examples of what each question was interrogating.

Case series

Author and year Country [Citation]	Clear inclusion criteria?	Reliable standard measurement of presenting condition?	Valid identification of infection (e.g. defined microbiology)?	Consecutive inclusion?	Complete inclusion and reporting?	Clear patient demographic data (e.g. range and mean of patient age)?	Clear clinical reporting (pre-treatment)?	Clear outcome or follow-up reporting (e.g. clinical outcome)?	Comments on adverse effects?	Clear site demographic (e.g. study location)?	Appropriate statistical analysis, if present?	Comments
Slopek *et al.* 1987 Poland [16]	No	No	No	Unclear	Unclear	No	No	No	Unclear	Yes	Yes	Although the number of patients of each age, sex and clinical condition were all mentioned in this study, it is unclear the ages of each individual case.
Aslam *et al.* 2020 USA [33]	Yes	Yes	Yes	Yes	Yes	Yes	Yes	Yes	Yes	Yes	N/A	
Petrovic Fabijan *et al.* 2020 Australia [36]	Yes	Yes	Yes	Unclear	Unclear	Yes	Yes	Unclear	Yes	Yes	Yes	
Rubalskii *et al.* 2020 Germany [37]	Yes	Yes	Yes	Yes	Yes	Yes	Yes	Yes	Yes	Yes	N/A	

Tkhilaishvili *et al.* 2022<br>Germany<br>[38]	Yes	Yes	Yes	Unclear	Unclear	Yes	Yes	Yes	Yes	Yes	N/A	
Onallah *et al.* 2023<br>Israel<br>[23]	Yes	Yes	Yes	Yes	Yes	Yes	Yes	Yes	Yes	Yes	N/A	
Green *et al.* 2023<br>USA<br>[22]	Yes	Yes	Yes	Unclear	No	No	Yes	Unclear	Yes	Yes	N/A	TAILφR had provided phages to 12 patients but only included 11 in this report, citing insufficient information about the 12th patient.<br><br>Case 11 was reported as both 'bacteria cleared' and an unfavourable microbiological outcome. It was unclear if the secondary infections reported for cases 9 and 11 were caused by the original organism.

Case reports

Author (year) [citation]	Were the patient's demographic characteristics clearly reported?	Was the patient's history clearly described?	Was the current clinical condition clearly described?	Were diagnostic tests or methods and results clearly described?	Were the treatment(s) or intervention(s) clearly described?	Was the post-intervention clinical condition clearly described?	Were any adverse or unanticipated events clearly described?
Duplessis *et al.* 2017<br>USA<br>[35]	Yes	Yes	Yes	Yes	Yes	Yes	Yes
Chan *et al.* 2018<br>USA	Yes	Yes	Yes	Yes	Yes	Yes	Yes

[34]							
Rojas *et al.* 2022 Germany [18]	Yes	Yes	Yes	Yes	Yes	Yes	Unclear
Grambow *et al.* 2022 Germany [19]	Yes	Yes	Yes	Yes	Yes	Yes	Yes
Püschel *et al.* 2022 Germany [21]	Yes	Yes	Yes	Unclear if phage sensitivity testing pro-/retrospective.	Yes	Yes	No
Racenis *et al.* 2023 Latvia [20]	Yes	Yes	Yes	Yes	Yes	Yes	Yes
Blasco *et al.* 2023 Spain [24]	Yes	Yes	Yes	Yes	Yes	Yes	Yes

Supplementary file S4. Data extracted from included reports.

Report details		Clinical details				Efficacy				Safety & adverse effects
Author (year), [citation], location, study type	No. of relevant reports and microbiology	Condition details	Phage sensitivity	Phages	Treatment schedule and route(s)	Outcome	Resolved	Improved	No response	
Slopek *et al.* 1987<br><br>[16]<br><br>Poland<br><br>Case series	7/550<br><br>5 of 7 cases were Staphylococcal monoinfection. 2 of 7 cases were polymicrobial infections caused by *Pseudomonas*, *Escherichia* and Staphylococci. All infections were resistant to antibiotics.	'Pyopericardium'; complications after operations on open heart and large vessels	No phage sensitivity testing reported. However, according to Slopek *et al.* 1983 [39], sensitivity was confirmed (results not shown).	No details. However, according to Slopek *et al.* 1983 [39], a library of 259 phages was available for use; the phage concentrations used were not specified. Crude phage lysates were used therapeutically.	10ml of phage was given orally three times daily (TDS) before a meal and after gastric neutralisation. Phages were also applied locally as required as moist applications. It was not clear if the patients received concurrent antibiotics.	'In 6 cases phage therapy eliminated the infection and in 1 marked improvement was observed with a tendency towards healing of the wounds'.	6	1	0	No comments specific to these patients.<br><br>General comment: 'side effects are rarely encountered'.
Duplessis *et al.* 2017<br><br>[35]<br><br>USA<br><br>Case report	1/1<br><br>Antibiotic refractory *Pseudomonas aeruginosa*.	A 2-year-old male DiGeorge syndrome patient with complex congenital heart disease and multiple cardiac interventions including aortic arch plasty, repair of ascending aortic pseudoaneurysm, atrial and ventricular	Sensitivity confirmed.	A two-phage cocktail (phages unspecified, US Navy phage collection).<br><br>The individual titres of each of the phages in the cocktail were not reported.	Intravenous administration of the phage cocktail at 3.5 × 10^5 plaque-forming units (PFU) every 6h. Dose was determined by the limit of endotoxin administration (5 EU/kg/h). The patient also received concurrent antibiotic therapy consisting of meropenem, tobramycin, and polymyxin B; regimens were not stated.	The patient tolerated the first 6 doses over 36h, after which phage therapy was stopped due to decompensation concerning for anaphylaxis, but subsequently attributed to progressive heart failure, although endotoxin release could not be excluded as a contributing factor.	0	1	0	Endotoxin release could not be excluded as a contributing factor to the patient's decompensation 36h after initial phage administration.

		septal defect closures, and pacemaker insertion. Antibiotic refractory *P. aeruginosa* infection and bacteraemia followed atrial and ventricular septal defect closures. Imaging showed infected fluid collections adjacent to the ascending aorta and cerebral mycotic aneurysms. Surgery deemed inappropriate due to risk of catastrophic bleeding.				Blood cultures taken on days 4 and 5 after phage therapy were sterile, but subsequent cultures after the cessation of phage therapy reverted to positive. Phage therapy was resumed 11 days after cessation, and within 1 day, blood cultures became sterile for several days which coincided with clinical improvement. Later deterioration refractory to subsequent interventions was attributed to the progression of undrained infectious fluid collections, antecedent influenza infection, and end-stage cardiac failure. The family elected to withdraw care, after which the patient died.				
Chan *et al.* 2018<br>[34]<br>USA<br>Case report	1/1<br>Antibiotic refractory *P. aeruginosa.*	76-year-old male with an aortic graft infection (aortic arch replacement with a Dacron graft).	Phage sensitivity not reported.	OMKO1 (Yale University, USA) in phosphate-buffered saline.	A single local administration of 10 ml of OMKO1 (1 x 10^7 PFU/ml) and ceftazidime (0.2 g/ml) was performed. The patient received concurrent ceftazidime; regimen was not specified.	Ceftazidime was stopped shortly after surgery. The patient did not show evidence of recurrent *P. aeruginosa* infection via blood cultures, clinically or via imaging.<br><br>Approximately 4 weeks post-procedure, an exploratory surgery was performed due to bleeding from the mediastinal fistula representing aortic perforation. Partial explantation of the graft and repair of the mediastinal fistula were undertaken, but not all of the graft could be removed due to the scarring and inflammation. Cultures taken only revealed	1	0	0	The day after the procedure 'the patient had no complaints, exhibited stable vital signs and had laboratory values within normal limits'.<br><br>'We did not observe any noticeable side effects with phage OMKO1'.

						growth of *Candida* in the superficial chest wound, which was treated.				
Petrovic Fabijan *et al.* 2020<br><br>[36]<br><br>Australia<br><br>Case series	8/13 Methicillin susceptible *S. aureus* (MSSA)<br><br>Antibiotic sensitivities not reported. Patients had at least two consecutive days of *S. aureus* bacteraemia.	65-year-old male with septic shock and prosthetic valve endocarditis (aortic, mitral).	A mixture of pro- and retrospective testing was used. It was not clear which approach was used for each patient.	AB-SA01 three-phage cocktail (AmpliPhi Biosciences, Australia).<br><br>Each of the three phages was present at 10^9 PFU/ml.	AB-SA01 (1 x 10^9PFU/ml; volume not specified) was administered intravenously in 50100ml of 0.9% NaCl over 10-30min twice daily (BD) for 14 days.<br><br>The patient received 28 doses of phage therapy. Antibiotics (flucloxacillin [2g every 4h {q4h}, 14g/24h], ciprofloxacin [400mg TDS], rifampicin [300mg BD] and meropenem [1g TDS]) were received before and during phage therapy and started up to 10 days before phage therapy.	Reduced CRP and white blood cell count (WBC) were observed by day 14 and the patient survived to follow-up at days 28 and 90. This patient avoided the need for valve surgery.	1	0	0	'No new fevers, rashes, hypotension or other adverse reactions were reported during and in the first 4h following AB-SA01 infusion (Supplementary Fig. 1) or attributed to AB-SA01 infusion by the care team at any other time. Thus, AB-SA01 seemed to be safe and the infusions well tolerated.'
		47-year-old male with septic shock and infective endocarditis (aortic, mitral).			AB-SA01 (1 x 10^9PFU/ml; volume not specified) was administered intravenously in 50-100ml of 0.9% NaCl over 10-30min BD for 14 days.<br><br>The patient received 5 doses of phage therapy. Antibiotics (flucloxacillin [2g four times daily {QID}]) were started 5 days before phage therapy.	The patient died during attempted surgical source control after only 48h of phage therapy, this followed 8 days of unremitting bacteraemic shock on optimal doses of antibiotics.	Insufficient evidence to draw a conclusion.			
		87-year-old male with possible prosthetic valve endocarditis (aortic).			AB-SA01 (1 x 10^9PFU/ml; volume not specified) was administered intravenously in 50-100ml of 0.9% NaCl over 10-30min BD for 14 days.<br><br>The patient received 4 doses of phage therapy. Antibiotics (flucloxacillin [2g QID], piperacillin-tazobactam [4.5g QID]) were received before and during phage therapy and started up to 8 days before phage therapy.	Phage therapy was withdrawn after 4 days when the decision was taken to move to palliative care, the patient then died on day 6. Retrospectively, *S. aureus* susceptibility to phage was noted to be low, with an efficiency of plating of <1.	Insufficient evidence to draw a conclusion.			
		36-year-old female with right-sided infective endocarditis (tricuspid).			AB-SA01 (1 x 10^9PFU/ml; volume not specified) was administered intravenously in 50-100ml of 0.9% NaCl over 10-30min BD for 14 days.<br><br>The patient received 28 doses of phage therapy. Antibiotics (flucloxacillin [2g q4h], clindamycin	This patient had two strains of *S. aureus*, one resistant to AB-SA01 and one susceptible. Reduced CRP and WBC were observed by day 14 and the patient survived to follow-up at days 28 and 90. Two	1	0	0	

					[450mg QID, 600mg TDS]) were received before and during phage therapy and started up to 7 days before phage therapy.	months later, the patient had *S. aureus* sepsis caused by a different sequence type; there was no evidence of the two initial infecting strains that were treated with phage therapy.				
		69-year-old female with prosthetic valve endocarditis (aortic, mitral).			AB-SA01 (1 x 10^9PFU/ml; volume not specified) was administered intravenously in 50-100ml of 0.9% NaCl over 10-30min BD for 14 days.<br><br>The patient received 28 doses of phage therapy. Antibiotics (flucloxacillin [2g q4h], ciprofloxacin [400mg BD], rifampicin [300mg TDS, 300mg BD], gentamicin [60mg TDS]) were received before and/or during phage therapy and started up to 8 days before phage therapy.	Reduced CRP and WBC were observed by day 14 and the patient survived to follow-up at day 28. The patient had valve replacement surgery more than 1 month after phage therapy when they were well and afebrile (on day 58 of antibiotics) and all resected tissues were culture-negative. Following elective surgery on day 58, the patient died suddenly on day 90.	1	0	0	
		21-year-old male with prosthetic valve endocarditis (aortic).			AB-SA01 (1 x 10^9PFU/ml; volume not specified) was administered intravenously in 50-100ml of 0.9% NaCl over 10-30min BD for 14 days.<br><br>The patient received 28 doses of phage therapy. Antibiotics (flucloxacillin [2g q4h, 12g/24h then 8g/24h], ciprofloxacin [400mg BD, then 400mg OD], rifampicin [300mg TDS, 600mg OD]) were received before and/or during phage therapy and started up to 6 days before phage therapy. The patient later received a second course of phage therapy, during which they continued to receive antibiotics.	This patient had not improved by day 14 and had culture-positive bacteraemia while receiving phage therapy, with no change in the phage susceptibility of their infecting *S. aureus*. This patient continued onto a second 14-day course of phage therapy, beginning on the day of prosthetic valve replacement surgery and following three weeks of antibiotics and the first two weeks of phage therapy. During the surgery, the resected paravalvular tissues were culture-negative, but *S. aureus* was detected in the valve tissues. The patient survived to day 90.	0	0	1	

		81-year-old male with prosthetic valve endocarditis (aortic).			AB-SA01 (1 x 10^9PFU/ml; volume not specified) was administered intravenously in 50-100ml of 0.9% NaCl over 10-30min BD for 14 days.<br><br>The patient received 27 doses of phage therapy. Antibiotics (flucloxacillin [2g q4h], ciprofloxacin [250mg BD], rifampicin [300mg BD]) were received during, and started 4 days before, phage therapy.	Reduced CRP and WBC were observed by day 14. The patient died from respiratory failure on day 27. Retrospectively, *S. aureus* susceptibility to phages was noted to be low, with an efficiency of plating <1.	Insufficient evidence to draw a conclusion.			
		70-year-old male with possible infective endocarditis (aortic).			AB-SA01 (1 x 10^9PFU/ml; volume not specified) was administered intravenously in 50-100ml of 0.9% NaCl over 10-30min BD for 14 days.<br><br>The patient received 28 doses of phage therapy. Antibiotics (flucloxacillin [2g q4h], cefazolin [2g TDS]) were received before and/or during phage therapy and started up to 4 days prior to phage therapy.	Reduced CRP and WBC were observed by day 14 and the patient survived to follow-up at days 28 and 90.	1	0	0	
Aslam *et al.* 2020<br><br>[33]<br><br>USA<br><br>Case series	4/10<br><br>Patients with antibiotic refractory infections caused by *P. aeruginosa* (n = 3) or *S. aureus* (n = 1).	60-year-old male with a ventricular assist device infection caused by *P. aeruginosa* and complicated by sternal osteomyelitis and recurrent bacteraemia.	Sensitivity confirmed.	Three-phage cocktail GD-1 (Adaptive Phage Therapeutics, USA).<br><br>The individual titres of each of the phages in the cocktail were not reported.	GD-1 (1.9x10^7 PFU/ml) was administered intravenously every 8h for 6 weeks. The patient also received concurrent antibiotics, regimen not specified.<br><br>After administration of the first dose in a clinical setting, with 2-3h adverse effect monitoring, the patient received self-administered phage therapy as an outpatient with weekly clinic visits.	The patient developed bacteraemia 1 week after starting phage therapy, which resolved with a change in antibiotics. Phage neutralising activity of the patient's serum was noted at this time. Recurrent driveline drainage developed after phage therapy.	0	0	1	'No phage-related adverse event'.

		65-year-old male with a ventricular assist device infection caused by *S. aureus* and complicated by sternal osteomyelitis and recurrent bacteraemia.		AB-SA01 three-phage cocktail (Armarta Pharmaceuticals, USA).<br><br>The individual titres of each of the phages in the cocktail were not reported.	AB-SA 01 (3×10^9 PFU/ml) was administered intravenously every 12h for 4 weeks. The patient also received concurrent antibiotics, regimen not specified.<br><br>After administration of the first dose in a clinical setting, with 2-3h adverse effect monitoring, the patient received self-administered phage therapy as an outpatient with weekly clinic visits.	The infection resolved and the patient underwent a successful heart transplant.	1	0	0	'No phage-related adverse event'.
		82-year-old male with a ventricular assist device infection caused by *P. aeruginosa* and complicated by recurrent bacteraemia.		Episode 1: SDSU1 (phages: PAK_P and E217) and SDSU2 (phages: PAK_P1 and PAK_P5) phage cocktails (San Diego State University, USA). PAK_P1 was also used alone.<br><br>Episode 2: PPM3 four-phage cocktail (Walter Reed Army Institute of Research, USA).<br><br>The individual titres of each of the phages in the cocktail were not reported.	Episode 1: The patient received an intraoperative dose of phage therapy followed by 2×10^5 PFU/ml administered intravenously every 8h for 6 weeks; phage cocktail was not specified and was presumed to be SDSU1. This was followed by 7.58×10^5 PFU/ml of phage PAK_P1 alone for 10 days; regimen not specified. This was followed by intravenous administration of SDSU2 every 12h for 3 weeks. The first two doses were given at 1×10^{11} PFU/ml and subsequently adjusted to 4×10^{10} PFU/ml. It was unclear whether the patient also received concurrent antibiotics.<br><br>Episode 2: 1×10^9 PFU/ml of PPM3 was administered intravenously every 12h for 4 weeks. It was unclear whether the patient also received concurrent antibiotics.<br><br>After administration of the first dose in a clinical setting, with 2-3h adverse effect monitoring, the patient received self-administered phage therapy as an outpatient with weekly clinic visits.	The patient developed recurrent bacteraemia within 1 week of completing treatment episode 1. Three and a half months later, the patient underwent treatment episode 2 but developed recurrent bacteraemia in week 4 while still on phage therapy.	0	0	1	The patient developed fever, wheezing and shortness of breath ~2h after each of the two first consecutive doses of SDSU2 at 1×10^{11} PFU/ml. This resolved with acetaminophen, solumedrol, albuterol nebulization, and diphenhydramine.<br><br>SDSU2 was subsequently well tolerated in a dose escalation study and continued to be used at 10^{10} PFU/ml. The endotoxin concentration of the original preparation was 4.3U (below the FDA limit of 5mg/kg/h). The authors hypothesised that the reaction may have been caused by additional pyrogens in the solution (e.g. from

										manufacturing) which were sufficiently diluted at the lower phage concentration that was well tolerated.<br><br>No adverse reactions were observed to the other phage preparations.
		64-year-old male with a recurrent bacteraemia and probable aortic graft infection caused by *P. aeruginosa*.		PPM2 three-phage cocktail (Walter Reed Army Institute of Research, USA).<br><br>The individual titres of each of the phages in the cocktail were not reported.	2.6 × 10^6 PFU/ml of PPM2 was administered intravenously every 12h for 6 weeks. The patient also received concurrent antibiotic therapy (ciprofloxacin); regimen not specified.<br><br>After administration of the first dose in a clinical setting, with 2-3h adverse effect monitoring, the patient received self-administered phage therapy as an outpatient during the COVID-19 pandemic with weekly telemedicine video visits.	The patient had negative blood cultures while receiving phage therapy and ciprofloxacin. Weekly surveillance blood cultures remained negative for 4 weeks with no recurrence for 12 weeks after completing phage therapy.	1	0	0	'No phage-related adverse event'.
Rubalskii *et al.* 2020<br><br>[37]<br><br>Germany<br><br>Case series	6/8<br><br>Various antibiotic refractory infections caused by *S. aureus, E. faecium, P. aeruginosa, E. coli.*	52-year-old male with a prosthetic infection after aortic arch replacement caused by *S. aureus*, *P. aeruginosa* and *E. faecium*.	Sensitivity confirmed.	*Staphylococcus* phage Sa30<br>*Staphylococcus* phage CH1<br>*Staphylococcus* phage SCH1<br>*Staphylococcus* phage SCH111<br>*Staphylococcus* phage Sa30<br><br>*Escherichia* phage ECD7<br>*Escherichia* phage V18	*Staphylococcus* phage CH1, *Enterococcus* phage Enf1, and *Pseudomonas* phages PA5 and PA10 were used at 1 x 10^8 PFU/ml. It was unclear is this was the titre of the cocktail or the individual phages in the cocktail.<br><br>Two days before surgery, 25ml of phages, 6ml of (240mg) gentamicin, and 20ml (350mg) of daptomycin were administered locally via a pigtail catheter and one 50ml dose of phages was administered orally. Intraoperatively, 25ml of phages was administered locally.<br><br>Twice daily intravenous antibiotic therapy before and during phage therapy was 2000mg cefepime,	'Bacteria were not detected for 16 days after the last phage application. Unfortunately, the patient developed a subsequent infection caused by *P. aeruginosa* and *E. coli'.* The patient died from this infection two months after first receiving phage therapy. It was not known if the *P. aeruginosa* isolate was the same as the first, but the antibiotic susceptibility patterns of the two isolates were different, 'which would suggest it was an independent infection'.	0	1	0	'We did not observe any major, minor, or unexpected side effects of phage therapy in our treated patients.'<br><br>Six of eight patients had elevated CRP levels shortly after phage therapy, which decreased over the next few days. This was attributed to either normal postoperative conditions or significant

				Pseudomonas phage PA5 *Pseudomonas* phage PA10<br><br>*Enterococcus* phage Enf1<br><br>The phages were sourced from the Gabrichevsky Institute, Russia.	500mg daptomycin, 600mg linezolid, and tobramycin to a blood concentration of 2mg/L.					bacterial lysis due to phage therapy.		
		59-year-old male with a chronic vascular graft infection after aortic arch replacement caused by *S. aureus*.			*Staphylococcus* phage CH1 was used at 1 x 10^9 PFU/ml.<br><br>Local administration of 20ml of phages was performed every 12h for 2 days via a chest tube.<br><br>Intravenous antibiotic therapy before and during phage therapy was 2000mg flucloxacillin QID and 600mg rifampicin BD.	*S. aureus* was not detected after phage therapy. A positron emission tomography-computed tomography (PET-CT) seven months after phage therapy showed no evidence of infection.	1	0	0			
		62-year-old male with a fulminant pleural empyema after left ventricular assist device (LVAD) implantation caused by *S. aureus*.			*Staphylococcus* phage CH1 was used at 1 x 10^9 PFU/ml.<br><br>Local administration of 20ml of phages was performed every 12h for 7 days via a chest tube.<br><br>Intravenous antibiotic therapy before and during phage therapy was 500mg daptomycin OD.	*S. aureus* was not detected after phage therapy. A PET-CT two months after phage therapy showed no evidence of infection. The patient died from transplant failure 20 months after phage therapy ended; this was not considered to be related to the resolved infection or phage therapy.	1	0	0			
		51-year-old male with a chronic LVAD infection caused by *S. aureus*. Colonisation of nose and throat observed.			*Staphylococcus* phages Sa30, CH1, SCH1, and SCH111 were used at 1 x 10^9 PFU/ml. It was unclear is this was the titre of the cocktail or the individual phages in the cocktail.<br><br>Phage therapy was administered once daily orally (10-20ml), intranasally (2ml), and locally (10ml, after flushing with antiseptics and antibiotics, for the nine days. Once-daily oral (10-20ml) and twice-daily local (10ml, after flushing with antiseptics and antibiotics) administration were continued for a further six days.<br><br>Intravenous antibiotic therapy before and during phage therapy was 500mg daptomycin once daily.	There was a 100x reduction in *S. aureus* in the drainage fluid and eradication was achieved in the nose and throat. There was no evidence of bacterial resistance to phage therapy or of anti-phage antibodies up to two weeks after phage administration. The patient was offered but declined surgical intervention to improve the delivery of phages to the site of infection. The patient died 1.5 months after starting phage therapy due to *S. aureus* sepsis.	0	1	0			

		45-year-old male with a repetitive treprostinil pump infection caused by *S. aureus*.			*Staphylococcus* phage Sa30 was used at 4 x 10^{10} PFU/ml.<br><br>Local administration of 4ml of phages mixed with fibrin glue was performed intraoperatively.<br><br>Oral antibiotic therapy before and during phage therapy was 375mg sultamicillin BD.	*S. aureus* was not detected after phage therapy. Observation of the pump 1.5 months after phage therapy showed no evidence of infection or remnants of fibrin glue.	1	0	0	
		66-year-old female with a sternal wall healing disorder after mitral valve replacement and aortocoronary bypass caused by *E. coli*.			*E. coli* phages ECD7 and V18 were used at 4 x 10^{10} PFU/ml. It was unclear is this was the titre of the cocktail or the individual phages in the cocktail.<br><br>Local administration of 4ml of phages mixed with fibrin glue was performed intraoperatively.<br><br>Oral antibiotic therapy before and during phage therapy was 600mg clindamycin TDS.	*E. coli* was not detected after phage therapy and the wound was completely healed.	1	0	0	
Tkhilaishvili *et al.* 2022<br><br>[38]<br><br>Germany<br><br>Case series<br><br>3/4 cases also reported elsewhere [29-31].	4/4<br><br>Various antibiotic refractory infections caused by *S. aureus, Cutibacterium acnes* and *P. aeruginosa.*	53-year-old male with a chronic recurrent LVAD driveline infection caused by *P. aeruginosa*.<br><br>Case also reported in Tkhilaishvili *et al.* 2021 [31]	Sensitivity confirmed.	A three-phage cocktail containing *Pseudomonas* phages PNM, 14/1, and PT07 (Queen Astrid Military Hospital, Belgium).	Preoperatively, the phage cocktail containing each of the three phages at 10^7 PFU/ml was administered as a 7hr intravenous infusion (10ml/h).<br><br>Intraoperatively, a 50ml loading dose of the phage cocktail (10^8 PFU/ml) was administered during surgical debridement.<br><br>Topical application of the phage cocktail (10^8 PFU/ml) during sterile dressing changes was performed every 12h for 5 days.<br><br>The patient also received concurrent antibiotics; intravenous ceftolozane/tazobactam (1.5g TDS) for 2 weeks followed by oral rifampicin (450mg BD) and sulfamethoxazole-trimethoprim (960mg BD) for a total of 6 weeks of antibiotics.	'After operation no *P. aeruginosa* could be isolated, only *S. haemolyticus* grew from the driveline. The wound healed and the patient was discharged after 14 days with oral antibiotics [rifampicin (450mg BD) and sulfamethoxazole-trimethoprim (960mg BD) to cover the *S. haemolyticus* infection] for six weeks. For *Pseudomonas* no oral antibiotic option was available.'<br><br>'Unfortunately, the patient died four months after [phage therapy] due to LVAD pump thrombosis. However, at hospital re-admission, the CT scan and	1	0	0	*Comments from Tkhilaishvili* et al. *2022:*<br><br>'Except nausea in 2 patients and mild elevation of liver functions in 1 patient, we did not observe any side effects of phage therapy in our treated cohort'.<br><br>'The therapy was generally well tolerated, although nausea and mild elevation of liver functions occurred, followed by a normalisation of

						surgical site showed no signs of infection with a sterile driveline exit site, blood cultures were negative and CRP level was within normal ranges'.				laboratory values after an [(intravenous) bacteriophages dose reduction. Therefore, it is difficult to determine if the continuation of intravenous BT with the same dosage would have deteriorated the liver functions or resulted in adaptation and resolution.' *Comments from Tkhilaishvili* et al. *2021:* 'After the third (intravenous) dose of bacteriophages, gamma-glutamyl transferase (GGT) and direct bilirubin slightly increased. However, after reduction of the (intravenous) bacteriophage doses (decreasing 0,5 log PFU/ml from the initial titre of each bacteriophages), no further elevations of liver disease indicators were observed.'
		41-year-old male with Marfan syndrome and a chronic recurrent CIED and carotid subclavian bypass infection caused by MSSA and *C. acnes*. Case also reported in Exarchos *et al.* 2019 [29].		PYO bacteriophage cocktail (active against multiple pyogenic bacterial species) and *Staphylococcal* bacteriophage Sb-1 (The Eliava Institute, Georgia).	Intraoperatively, 10 ml of a mixture containing PYO bacteriophage cocktail (10^6 PFU/ml; titres of individual phages not specified) and Sb-1 (10^7 PFU/ml) at a 1:1 ratio was applied to the debridement site. No antiseptics were used during surgery. After surgery, a drain was placed into the surgical site before wound closure through which 5ml of the phage mixture was instilled every 8h for 14 days. Concurrent antibiotic therapy was intravenous flucloxacillin (2g/4h) and fosfomycin (5g/8h) for a total of 21 days, switched thereafter to oral levofloxacin (500mg/12h) and rifampicin (450 mg/12h) for 3 months.	'At follow-up after 3, 6, and 12 months, no local or systemic signs of infection were observed. Blood cultures remained negative, serum [CRP] was low, and the 3 months PET-CT showed reduced glucose uptake.'	1	0	0	
		67-year-old male with an abscess at a thoracotomy scar site with involvement of a LVAD caused by MSSA. Case also reported in Mulzer *et al.* 2019 [30].			Intraoperatively, 10 ml of a mixture containing PYO bacteriophage cocktail (10^6 PFU/ml; titres of individual phages not specified) and Sb-1 (10^7 PFU/ml) at a 1:1 ratio was applied to the debridement site. No antiseptics were used during surgery. After surgery, a drain was placed into the surgical site through which 5ml of the phage mixture was instilled every 8h for 10 days. Concurrent antibiotic therapy was intravenous meropenem (1g/8h) and f19osfomycin (5g/8h) followed by oral ciprofloxacin (750mg/12h) and rifampicin (450mg/12h) for a total of 3 months.	'The wound healed and the patient was discharged after 16 days. At the follow-up visit 9 months later, the surgical site showed no local signs of infection.'	1	0	0	

		68-year-old male with a chronic LVAD driveline infection caused by *P. aeruginosa*.		Autophage (assumed to be *Pseudomonas* phage selected for activity against the patient's isolate) and PYO bacteriophage cocktail (active against multiple pyogenic bacterial species; the Eliava Institute, Georgia).	Autophage (10^8 PFU/ml) was applied locally with a sterile dressing change every 12h for 12 days. PYO bacteriophage cocktail (10^6 PFU/ml; titres of individual phages not specified) was applied locally with a sterile dressing change every 12h for 5 days.<br><br>The patient also received intravenous meropenem (1g/8h) and gentamicin (120mg/day) followed by oral doxycycline (100mg/12h) and local gentamicin ointment for a total duration of 5 weeks. The relative timings of antibiotics and phages were not reported.	The infection relapsed and a phage-resistant *Pseudomonas* emerged. This was 'explained by complications of delivery of bacteriophages locally to all LVAD driveline infected sites during the sterile dressing changes.'	0	0	1	
Grambow *et al.* 2022<br><br>[19]<br><br>Germany<br><br>Case report	1/1<br><br>Antibiotic refractory MSSA.	67-year-old female with a TEVAR graft infection caused by MSSA.	Phage sensitivity not reported.	SniPha 360 (phage24.com, Austria), a phage cocktail active against *S. aureus, E. coli, P. Aeruginosa, Streptococcus pyogenes, Proteus vulgari,s* and *P. mirabilis*.<br><br>The individual titres of each of the phages in the cocktail were not reported.	A three-step approach was taken:<br><br>1) Extravascular treatment: left-sided thoracotomy, debridement, and jet-lavage, followed by 20ml of SniPha 360 (concentration not reported, diluted in 100ml NaCl) was applied to infected para-aortic tissue. Two endosponges were placed surrounding the arch and proximal descending aorta with VAC GRANFOAM dressing sponge connected to VAC VERAFLO system and the thorax closed. Once a day, intrathoracic fluid was pumped out via VAC system 2h prior to bacteriophage treatment, followed by flushing and draining of the sponges three times with 500ml 0.9% NaCl. Afterwards the endosponges were flushed with 20ml of SniPha 360 diluted in 100ml 0.9% NaCl.<br><br>2) A repeat thoracotomy was performed three days later. The sponges were removed and aortic and surrounding tissues were covered with 40 ml SniPha 360 in 15.8% hydroxycellulose gel.<br><br>3) Three days later, two sterile stent-grafts were externally coated with a mixture of 40ml SniPha	'PET-CT scans performed at three months and one year after the bacteriophage treatment did not reveal any signs of infection in or around the thoracic aorta. The patient recovered further, while infection parameters were undetectable without continuing antibiotic treatment.'	1	0	0	'No side effects, including immunological reactions after bacteriophage treatment, were observed.'

					360 and 15.8% hydroxycellulose gel and the grafts were implanted re-lining the existing graft.<br><br>Concurrent meropenem and cefazolin (regimens not stated) were discontinued after 12 days.				
Rojas *et al.* 2022<br><br>[18]<br><br>Germany<br><br>Case report	1/1<br><br>Antibiotic refractory *S. aureus*	49-year-old male with an outflow graft and intra-thoracic LVAD driveline infection caused by *S. aureus*.	Phage sensitivity not reported.	SniPha 360, (Sanubiom GmbH, Austria) a phage cocktail active against *S. aureus, E. coli, P. aeruginosa, Streptococcus pyogenes, P. vulgaris* and *P. mirabilis*.<br><br>The individual titres of each of the phages in the cocktail were not reported.	Wound preparation was redo sternotomy, intra-operative microbiology, debridement, washout with pulse lavage and antiseptic, and VAC therapy applied (Veraflo) with instillation therapy (Lavanox, instilled for 10 minutes every 4h for 19 days).<br><br>Wound debridement and lavage were performed again with antiseptic and NaCl. Following this, 20ml of SniPha 360 ('1 x 10^7 CFU/ml') was applied using a novel viscous injection media and the wound was closed. The patient received concurrent piperacillin/tazobactam (4.5g TDS).	After an 'uneventful' 11 days, the patient was discharged from hospital. A three-month follow-up PET-CT demonstrated a significant reduction in signs of infection around the outflow graft and intra-thoracic driveline.<br><br>Six months later, the patient was admitted with a superficial infection at the driveline exit site (*S. aureus*), but this was not felt to be related to the previous intra-thoracic septic focus. This was managed with local treatment with disinfectants and wound dressings; no systemic antibiotics were given.	1	0	0	No comment.
Püschel *et al.* 2022<br><br>[21]<br><br>Germany<br><br>Case report	1/1<br><br>*P. mirabilis* and *S. aureus*.	57-year-old male with an LVAD driveline infection.	Sensitivity confirmed, unclear if pro- or retrospectiv e.	SniPha 360, (Sanubiom GmbH, Austria) a phage cocktail active against *S. aureus, E. coli, P. Aeruginosa, Streptococcus pyogenes, P. vulgaris,* and *P. mirabilis*.<br><br>The individual titres of each of	Debridement, jet lavage with antiseptic and driveline coating with Gore® Synecore were performed. Then, 20ml of SniPha 360 ('1 x 10^7 CFU') was diluted (dilutions not specified) in saline and polysaccharide (Starsil, Hemostat Manufacturing GmbH, Germany). The resulting viscous fluid was applied between the driveline and Synecore coating. The phage-containing fluid was also applied to subcutaneous tissue surrounding the driveline before wound closure.<br><br>The patient had received empirical piperacillin/tazobactam (4.5g TDS) on admission, to which both bacterial species were sensitive. It was	The patient had uncomplicated wound healing, and antibiotic therapy was switched to oral cotrimoxazole, to which both the *P. mirabilis* and *S. aureus* were sensitive (dose and timing of the switch not specified) and the patient was discharged after 20 days with primary wound healing.<br><br>Two months later, the patient was readmitted with a mild local *S. aureus* infection of the	1	0	0	No comment.

				the phages in the cocktail were not reported.	not explicit whether this was continued during phage therapy.	driveline exit. 'Long time calculated' antibiotic therapy with flucloxacillin was initiated and the patient discharged after 14 days. At follow up 8 months later, there were no signs of local or systemic infection.				
Racenis *et al.* 2023<br><br>[20]<br><br>Latvia<br><br>Case report	1/1<br><br>Antibiotic refractory *P. aeruginosa.*	54-year-old male with an LVAD driveline infection caused by *P. aeruginosa*.	Sensitivity confirmed.	A two-phage cocktail containing *Pseudomonas* phages PNM and PT07 (Queen Astrid Military Hospital, Belgium).	The phage cocktail (PNM and PT07 each at 10^7 PFU/ml) was administered intravenously 2h before surgery using an infusion pump (13ml/h for 6h through a central venous catheter for a total volume of 80m). This was repeated once daily for 8 days.<br><br>The surgical intervention consisted of extensive tissue debridement with local antiseptic treatment with Prontosan®. A new subcutaneous canal was prepared for driveline repositioning. The new and previous canals were then irrigated with 250ml of 0.9% NaCl followed by 250ml of 4.2% $NaHCO_3$. Five minutes later, 50ml of phage cocktail containing was applied to the wound. The driveline was repositioned and phage administered along the driveline using an 8-Fr catheter. The wound was left open for healing.<br><br>The day after surgery and for a total of three days, the wound was rinsed via the 8-Fr catheter with 50ml of phage cocktail. Prior to phage administration, the wound was rinsed with 250ml 0.9% NaCl and then with 250ml 4.2% $NaHCO_3$.<br><br>The patient received intravenous colistin (3 million IU TDS) until the surgical intervention. Concurrent intravenous antibiotic therapy was administered from 2h before surgery, consisting of ceftazidime/avibactam (2.5g TDS) and amikacin	Dressing changes and swabbing were performed daily and did not show *P. aeruginosa*. Six days after surgery, the secondary healing wound did not show signs of infection and was closed. The patient was discharged 45 days after surgery, following an 18-F-FDG PET-CT scan which did not show signs of ongoing infection and with both CRP and WBC within the normal range. A follow-up PET-CT scan at 34 weeks showed no signs of significant metabolic activity. The patient had no signs of recurrence at 21 months after treatment.	1	0	0	'During phage treatment, no adverse events were observed'.<br><br>The case 'shows that intravenously applied phages in concentration 10^7 were safe and did not elicit side effects'.

					(750mg BD). Amikacin was continued for 4 weeks and ceftazidime/avibactam for 6 weeks.					
Onallah *et al.* 2023<br><br>[23]<br><br>Israel<br><br>Case series	1/20<br><br>Antibiotic refractory *P. aeruginosa.*	A 10-year-old female with a *P. aeruginosa* Berlin heart (ventricular assist device) infection.	Sensitivity confirmed.	Pa14NϕPASA16 (Adaptive Phage Therapeutics, USA).	Case information provided as part of a larger mixed case series. No clinical details specific to this case were provided, aside from the noting that the patient received concurrent meropenem.	'Treatment failure' was recorded but no details were provided at the 2-month follow up. Case report was in preparation at the time of this review.	0	0	1	No comment specific to this patient. General comment in this case series: 'no major side effects were reported by the patients'.
Blasco *et al.* 2023<br><br>[24]<br><br>Spain<br><br>Case report	1/1<br><br>Antibiotic refractory *P. aeruginosa*	An axillo-bifemoral bypass prosthetic vascular graft infection with recurrent bacteraemia caused by *P. aeruginosa*.	Sensitivity confirmed.	A three-phage cocktail containing *Pseudomonas* phages PNM, 14/1, and PT07 (Queen Astrid Military Hospital, Belgium).	The phage cocktail was administered intravenously (each phage at 10^7 PFU/ml ; total volume of 70ml; giving a daily dose 2.1 x 10^9 PFU) OD as a 6h infusion for 7 days.<br><br>The patient received ceftazidime-avibactam for 6 weeks before phage therapy and for 2 weeks after (regimen not specified) as well as amikacin during phage therapy (regimen not specified).	The month after receiving phage therapy, and in the absence of antibiotic therapy, the patient experienced *P. aeruginosa* bacteraemia. Sequencing showed this *P. aeruginosa* strain to be more susceptible to beta lactams and quinolones. The patient underwent surgery for graft replacement, followed by antibiotics and remained asymptomatic 10 months after surgery.	0	0	1	'No adverse events were observed.'
Green *et al.* 2023<br><br>[22]<br><br>USA<br><br>Case series	3/12<br><br>Antibiotic refractory *S. aureus* (n = 2) and *P. aeruginosa* (n = 1).	An LVAD-related infection with recurrent bacteraemia caused by *S. aureus*.	Sensitivity confirmed.	A two-phage cocktail containing phages K and SA4 (Baylor College of Medicine, USA).<br><br>The individual titres of each of the phages in the cocktail were not reported.	The phage cocktail was administered as a single intraoperative dose (3 x 10^{10} PFU/ml; volume not specified) and intravenously (10^{10} PFU/ml) BD for 6 weeks.<br><br>The patient received concurrent ceftriaxone (regimen not specified).	'Favourable outcome: 19 months after completing phage therapy the patient successfully underwent a heart transplant and all antibiotics for the infection were stopped.'	1	0	0	'No adverse effects.'
		An LVAD-related infection with recurrent bacteraemia and ongoing device-			The phage cocktail was administered intravenously (10^9 PFU/ml) BD for 6 weeks.<br><br>The patient received concurrent cefazolin and ertapenem (regimens not specified).	'Unfavourable outcome: bacteraemia recurred after the end of phage and antibiotic therapy. He remains on suppressive antibiotics to date.'	0	0	1	

		associated abscess caused by *S. aureus*.								
		A persistent LVAD-driveline infection caused by *P. aeruginosa*.		A two-phage cocktail containing phages 6917 and 6959 (Baylor College of Medicine, USA).<br><br>The individual titres of each of the phages in the cocktail were not reported.	The phage cocktail was administered as a single intraoperative dose (3 x 10^{11} PFU/ml; volume not specified) and intravenously (10^{10} PFU/ml) BD for 6 weeks.<br><br>The patient received concurrent cefepime (regimen not specified).	'Favourable outcome: patient continues to have scant drainage culture-positive for *P. aeruginosa* that is controlled with local wound care. CT imaging shows no evidence of infection. Currently the patient is off antibiotic therapy since May 2022 and stable.'	1	0	0	
